# Prediction approach of larch wood density from visible–near-infrared spectroscopy based on parameter calibrating and transfer learning

**DOI:** 10.3389/fpls.2022.1006292

**Published:** 2022-10-04

**Authors:** Zheyu Zhang, Yaoxiang Li, Ying Li

**Affiliations:** ^1^ College of Engineering and Technology, Northeast Forestry University, Harbin, China; ^2^ College of Energy and Transportation Engineering, Inner Mongolia Agricultural University, Hohhot, China

**Keywords:** visible and near-infrared spectroscopy, calibration transfer, transfer learning, larch, wood density

## Abstract

Wood density, as a key indicator to measure wood properties, is of weighty significance in enhancing wood utilization and modifying wood properties in sustainable forest management. Visible–near-infrared (Vis-NIR) spectroscopy provides a feasible and efficient solution for obtaining wood density by the advantages of its efficiency and non-destructiveness. However, the spectral responses are different in wood products with different moisture content conditions, and changes in external factors may cause the regression model to fail. Although some calibration transfer methods and convolutional neural network (CNN)-based deep transfer learning methods have been proposed, the generalization ability and prediction accuracy of the models still need to be improved. For the prediction problem of Vis-NIR wood density in different moisture contents, a deep transfer learning hybrid method with automatic calibration capability (Resnet1D-SVR-TrAdaBoost.R2) was proposed in this study. The disadvantage of overfitting was avoided when CNN processes small sample data, which considered the complex exterior factors in actual production to enhance feature extraction and migration between samples. Density prediction of the method was performed on a larch dataset with different moisture content conditions, and the hybrid method was found to achieve the best prediction results under the calibration samples with different target domain calibration samples and moisture contents, and the performance of models was better than that of the traditional calibration transfer and migration learning methods. In particular, the hybrid model has achieved an improvement of about 0.1 in both *R* ^2^ and root mean square error (RMSE) values compared to the support vector regression model transferred by piecewise direct standardization method (SVR+PDS), which has the best performance among traditional calibration methods. To further ascertain the generalizability of the hybrid model, the model was validated with samples collected from mixed moisture contents as the target domain. Various experiments demonstrated that the Resnet1D-SVR-TrAdaBoost.R2 model could predict larch wood density with a high generalization ability and accuracy effectively but was computation consuming. It showed the potential to be extended to predict other metrics of wood.

## 1 Introduction

Wood density is an important physical property to test the mechanical properties of wood (Li et al., 2019), and it is also an important indicator to identify the quality of wood (Thomas et al., 2009). From the perspective of forestry, wood density can be used to predict the physical and mechanical properties of wood, such as hygroexpansion, hardness, and strength (Missanjo and Matsumura, 2016). Accurate prediction and evaluation of wood properties can provide the theoretical and scientific bases for many aspects such as material improvement, plantation cultivation, improvement of the comprehensive utilization rate of wood, and restoration and maintenance of wood-frame buildings (Fukatsu et al., 2013; Francis et al., 2017; Alade et al., 2022). Therefore, rapid and accurate acquisition of wood density is of great significance to modern forestry production. Traditional wood density detection methods include the drainage method, weighing method, and mechanical force-based density detection method (Alves et al., 2020). However, the processes of the above methods are complicated and time-consuming, which are not conducive to the density testing of large quantities of wood in practice. Visible–near-infrared (Vis-NIR) spectroscopy records the combination vibrations of hydrogen-containing groups at the molecular level of samples (Benedet et al., 2020), which can be combined with chemometric techniques for rapid, non-destructive qualitative and quantitative analyses of wood properties (Chen and Li, 2020). It provides an efficient and feasible solution for the real-time determination of wood density. However, many obstacles still exist in the practical estimation of wood density by spectral non-destructive testing (NDT) methods, such as high collinearity of spectral data, oversensitivity of spectra to instruments and environments, and poor predictive performance of the models. Overcoming these obstacles has also become a research priority in chemometrics.

In recent years, deep learning (DL) methods have been favored by many researchers in the field of spectroscopy, mainly because DL has obvious advantages in solving high-dimensional spectral data as a deep non-linear network mapping structure model (Cai et al., 2022). There are hundreds or thousands of characteristic wavelengths in a spectrum, and spectral features can be excavated and learned from superficial to in-depth and layer-by-layer by DL, which is similar to imitating the thinking mode of the brain (Ghosh et al., 2019). Multi-layer neural networks, as a common form of DL, can realize end-to-end non-linear mapping of spectral data; thereby, abstract features in spectra are simplified, and complex classification and regression problems in spectra are realized (Sommers et al., 2020).

In the field of agriculture and forestry, the application of spectroscopy has become a research boom combined with DL gradually (Chen et al., 2016; Kawamura et al., 2021; Qiao et al., 2021). In the detection and adjustment of forest resources, we can grasp the dynamic pattern of forest resources in time while macro-regulating the state of economic management. Distinguishing tree species with different economic values has great potential by combining airborne hyperspectral remote sensing technology with DL. Trier et al. (Trier et al., 2018) employed convolutional neural network (CNN) to classify the Vis-NIR spectral channels of the main tree species in the Norwegian forest, resulting in good classification rates. Mayra et al. (Mayra et al., 2021) proposed 3D-CNN combined with hyperspectral remote sensing to identify a variety of major tree species in Finland accurately. Identifying the quality of agroforestry economic products rapidly can improve the quality of the products, by assisting manufacturers in adjusting their cultivation programs in a timely manner, during the cultivation process (Assadzadeh et al., 2020). The flaw detection, pesticide detection, and species identification of agricultural and forestry products can promote the rapid development of the entire production chain (Jin et al., 2018; Feng et al., 2019; Zhang et al., 2020a).

However, the optical measurement signal is disturbed by the type of instrument, detection principle, and detection environment (temperature, humidity, noise, etc.) greatly, leading to large deviations in results and poor model applicability, making it difficult for spectroscopic techniques to be widely used. Calibration transfer is one of the effective methods to solve this technical problem (Qin and Gong, 2016). The generalization ability of the model can be improved by calibration transfer from two perspectives. One is exploring the linear relationship between master and slave models to improve the adaptability of the models themselves; the other is correcting different data domains through statistical methods or chemometric methods to eliminate the deviations between different data domains as much as possible. In the first perspective, slope/bias correction (SBC) (Bouveresse et al., 1994) is typical. In the second perspective, various methods such as piecewise direct standardization (PDS) (Wang et al., 1991), spectral space transformation (SST) (Du et al., 2011), and canonical correlation analysis (CCA) (Fan et al., 2008) are applied widely. Many investigations have indicated that the generalization ability of the model is improved and the discrepancies between different data fields are ameliorated by applying the above methods, but the results are uneven, and most of them are not ideal. Most calibration transfer methods are limited by data dimension and sample size and cannot deal with related issues flexibly.

In the field of DL, researchers have discovered a concept similar to calibration transfer called transfer learning (TL) (Sun et al., 2019). Analogously, the master model in the calibration transfer corresponds to the source domain in TL, and the slave model in the calibration transfer corresponds to the target domain in TL. The core of TL is to find the similarity between known and unknown domains and apply the knowledge and laws to the unknown domain learned in the known domain (Larsen and Clemmensen, 2020). The theory of global sharing of model parameters in DL is consistent with TL, and the shortcomings of “dimensional disaster” in high-dimensional data can be solved by deep neural networks (Johnstone and Titterington, 2009), so deep transfer learning has developed rapidly in the field of spectroscopy. In agriculture and forestry, applications of transfer learning include the following: first, the most common application was the identification of tree species, including the rapid identification of economical woods (Li et al., 2022), pests, and quality defects (Chen et al., 2020; Ahmad et al., 2021; Alencastre-Miranda et al., 2021). Second, TL was used for forest and farmland management and ecosystem status assessment (Astola et al., 2021; Jin et al., 2021). Third, TL is used for the prediction of the properties of wood and agricultural products (Singh et al., 2021).

In CNN-based transfer learning, using a pretrained network to initialize the network parameters of any layer and constraining the parameter changes with a smaller learning rate (fine-tuning) (Shin et al., 2016) and fine-tuning only the weights of the final fully connected layer of the network (feature extractor) (Gao and Mosalam, 2018) are two common application scenarios. In particular, the classifier can be modified or added after the pretrained network during feature extraction to make it a feature extractor for the target domain (Li et al., 2020a).

In the CNN extraction of neck features, each convolution kernel is acted as a filter to perform convolution operations, and the weights of features are reassigned according to the layer-by-layer recognition of the convolution kernels, thereby increasing the separability of linearly inseparable datasets (Mei et al., 2017). The activation function of CNN (such as Softmax and ReLU) performs macro-control on the feature weights (Roy et al., 2020). In this process, the samples with the Intersection-over-Union (IoU) greater than 0.5 are marked as positive samples by CNN and vice versa as negative samples (Cai and Vasconcelos, 2021). Usually, CNNs require a large number of samples, and correspondingly, the prediction accuracy of small sample data (e.g., spectral data) significantly declines. Support vector machine (SVM) is different from the principle of CNN, which maps non-linear features into high-dimensional space to achieve classification (increasing IoU). Some studies have proved that using SVM as the classifier of CNN (CNN-SVM) can improve the prediction ability of CNN for small sample datasets (Niu and Suen, 2012). For regression problems, a support vector regression machine (SVR) is used as a regressor of CNN (Zhou et al., 2021).

Although the risk of overfitting CNN models can be reduced by CNN-SVM, in the TL domain, CNN-SVM also lacks the ability to adjust the sample weights in the source and target domains dynamically when the two vary greatly. At the same time, CNN-SVM cannot update the hyperplane division rules in time, which lacks flexibility in the face of unpredictable external disturbances in actual production. In summary, this study took larch wood density as the research object and aimed to propose a parameter-calibrated transfer learning method to predict wood density under different moisture contents. The deep Resnet network is used for the first time to construct a Vis-NIR spectral model, and SVR is used as a regressor for the network to accommodate spectral datasets with small sample sizes. At the same time, the algorithm attempts to achieve automatic calibration of sample parameters depending on whether the contribution values of their weights are positive or negative during the iterative process. The hybrid model validates the feasibility and potential of deep migration learning strategy in quantitative spectral analysis and explores the application of machine learning in the direction of wood non-destructive testing.

This paper is organized as follows. Section 2 details the larch air-density measurements and spectra under different moisture content conditions used in this study and the proposed Resnet1D-SVR-TrAdaBoost.R2 hybrid model; the prediction results of different calibration transfer and transfer learning methods, the validation of the target domain correction sample size, and the performance of different moisture content correction transfer models are presented in Section 3; the maximum iteration number of iterations on model performance and the application of hybrid models in forestry are discussed in Section 4; the results of the study are summarized in Section 5.

## 2 Methods and materials

### 2.1 Description of proposed models

The model proposed in our paper is Resnet1D-SVR-TrAdaBoost.R2, which consists of two parts chiefly: one is the Resnet-SVR used for building the transfer model, and the other is the TrAdaBoost.R2 used for parameter calibrating.

The core principle of CNN is to learn the mapping relationship between input and output (Aslam et al., 2021). It avoids explicit feature extraction and learns implicitly from the mapping relationship in the data when used as a feature extractor. As a kind of one-dimensional (1D) input data, Vis-NIR spectral data have the disadvantages of high collinearity and spectral peak overlap (Li et al., 2020a), so increasing the network depth is beneficial to extract more effective spectral features. Meanwhile, to avoid the problem of network degradation, Resnet is chosen as the feature extractor. The residual building block is a shortcut connection and a key part of Resnet, which helps to avoid the gradient explosion/vanishing problem during the back-propagation of errors, thereby improving the robustness of deep network models (Wen et al., 2020).

**Figure 1:**
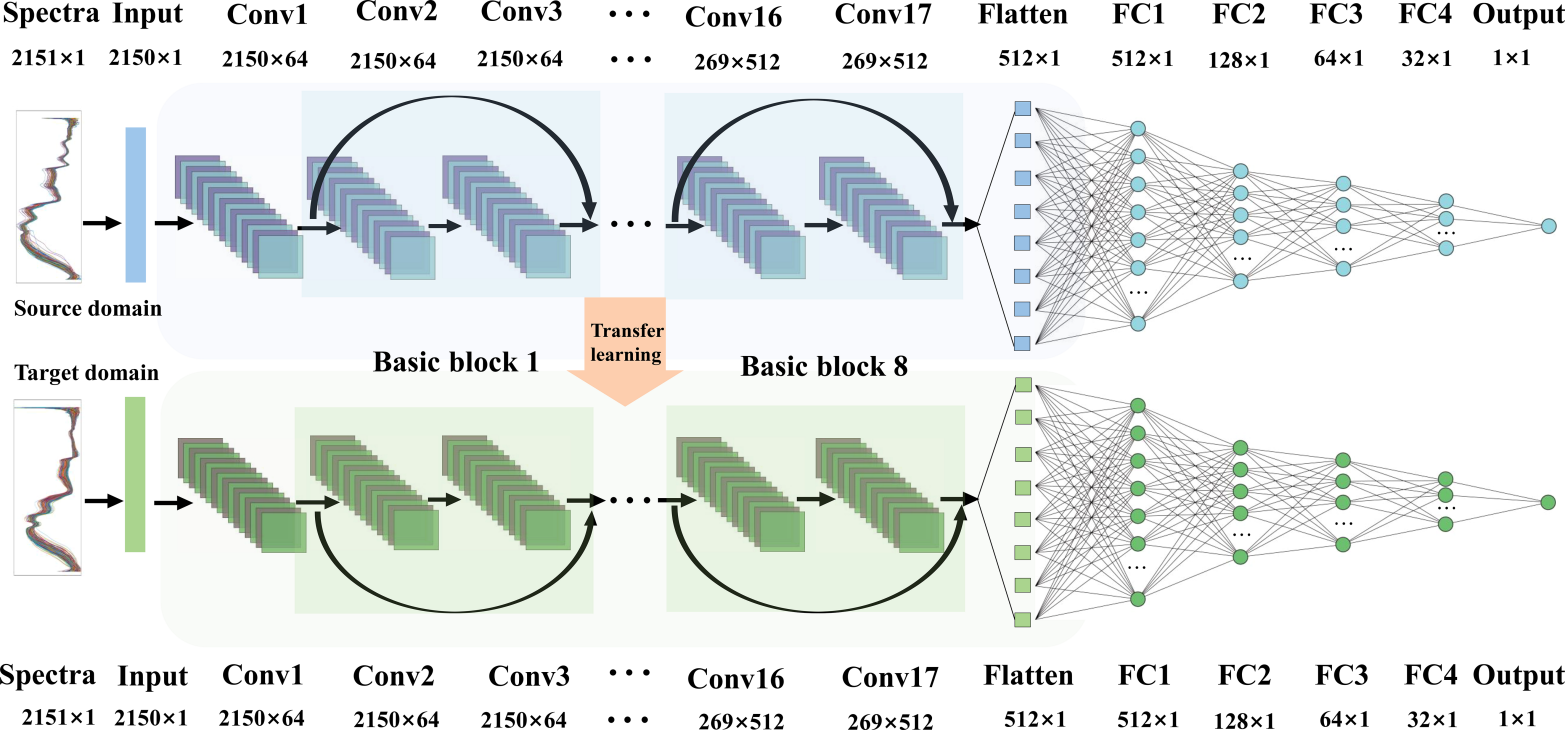
Flowchart of the 1D Resnet architecture.

A deep 1D Resnet model is constructed in this study to process the Vis-NIR spectral 1D data, which includes an input layer, and four residual building blocks; after being flatten, the features are followed by four fully connected (FC) layers with sizes of 512, 128, 64, and 32 and an output layer (**Figure 1**). Each residual building block consists of two basic blocks, each of which consists of two convolutional layers (Conv), a batch normalizations layer (BN), and a shortcut. The size of the convolution kernel is 3, and the number of convolution channels is set as 64, 128, 256, and 512 in ascending order. The activation function of each layer except the output layer is set as the rectified linear element function (ReLU), and the activation function of the output layer is set as a linear function (Linear) to make the network a regression model. Adam optimizer is used for training by the proposed model (Bera and Shrivastava, 2020). In order to speed up the convergence of training data and reduce the amplitude of training vibrations, the batch size is determined to be 5. The mean square error (MSE) is used as the loss function of Resnet, and then, the coefficient of determination (*R* ^2^) and the mean absolute error (MAE) are selected as the evaluation metrics of the model. In addition, the ReduceLROnPlateau function and EarlyStopping function provided by Keras are introduced to avoid the model falling into the local optimum.

According to the nature of convolution and pooling computation, it can eliminate the influence of the spectral feature drift part on the selected feature vector and reduce the risk of overfitting. The fully connected layer of CNN can be considered as a linear classifier operator for the features extracted by the previous convolutional layer (Zhang et al., 2020b). The values output *via* the Flatten layer already contain features of the spectrum, and it is feasible to consider these output features as inputs to other regression methods for analysis (Li et al., 2020a). Since the high prediction accuracy of the CNN model is based on large sample size, in spectral analysis problems, the number of wavelength variables often far exceeds the number of samples. Therefore, the hybrid Resnet-SVR model is proposed to improve the learning ability for small samples and solve the tough problem of the application of spectral quantitative analysis in traditional DL.

**Figure 2:**
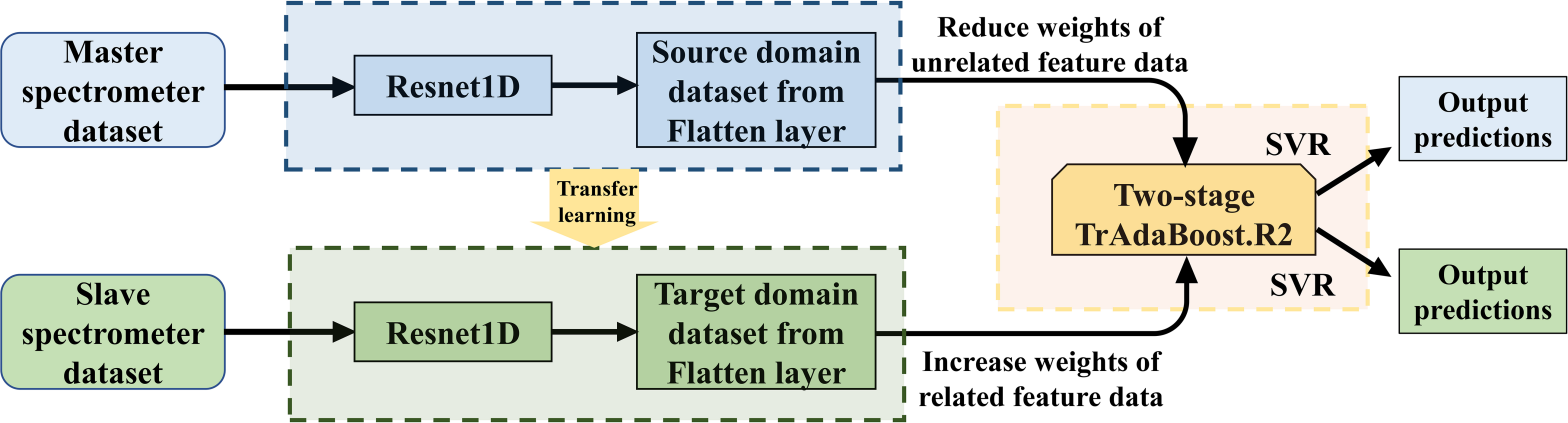
Schematic diagram of Resnet1D-SVR-TrAdaBoost.R2.

In the basic process of Resnet-SVR, there are two main steps: first, the preprocessed spectral dataset is fed input to the proposed Resnet model for pretraining, and second, the features extracted by Resnet are input to the SVR for training and evaluation (**Figure 2**). Among them, the kernel function of SVR is determined as radial basis function (RBF), and the hyperparameters of SVR (penalty factor C, kernel parameter gamma, and kernel width epsilon) are optimized using particle swarm optimization (PSO) algorithm to achieve the optimal regression effect (Han et al., 2021). In the PSO, the population size is set to 50, the individual learning factor c1 = 1.5, the social learning factor c2 = 1.7, the maximum number of iterations is set to 50, and the cross-validation fold is set to 10-fold.

Transfer learning is a type of machine learning method that uses the knowledge learned previously to solve problems in new fields more quickly for similar fields. Among them, the transfer of features and models is used in current research widely. The general idea of transfer learning in this study can be summarized as extracting similar features to minimize the differences in related domains and then developing models to find parameters shared between related domains to reduce the demand for target domain data, while the parameters are calibrated with the aim of adapting the model to the target domain, thereby improving the learning effect. Recently, with the popularity of deep learning methods, deep neural network models with characteristics of global weight sharing have also been used in transfer learning (deep transfer learning) gradually, which can extract more expressive features automatically, and therefore applied to computer vision, text dataset processing, and voice or audio recognition widely (Zhang et al., 2020c).

TrAdaBoost is a traditional transfer learning framework (Yehia et al., 2021). TrAdaBoost assumes that the input features and output labels of the source and target domains with different distributions are the same and assigns an initial weight to each input sample. In each round of iteration, the weight of target domain samples that are misclassified will be increased, and the weight of source domain samples that are misclassified will be decreased, which is the same as the strategy of AdaBoost to update weight (Yu et al., 2021). Two-stage TrAdaBoost.R2 is an extension of AdaBoost.R2 (Li et al., 2021) for solving regression problems of TL, which can solve the problem of negative transfer of source and target domains. In the first stage, when the weights of the target domain tend to reach zero, binary search is used to replace the error rate to update the weights of the source domain. In the second stage, the weights of source domain weight are fixed, and AdaBoost.R2 is called to update the weights of the target domain. The details of the two-stage TrAdaBoost are described in **Table 1**.

**Table 1:** Detailed steps of Resnet1D-SVR-TrAdaBoost.R2.

Calibration transfer Resnet1D-SVR-TrAdaBoost.R2.
**Input**: Source domain dataset $\left\{{\text{X}}_{s}^{\text{i}},{\text{Y}}_{s}^{\text{i}}\right\}(i=1,2,\ldots ,\text{m})$ and target domain dataset $\left\{{\text{X}}_{t}^{\text{i}},{\text{Y}}_{t}^{\text{i}}\right\}(\text{i}=1,2,\ldots ,\text{n})$ ; The number of frozen layers **L**; The value of maximum iterations **N**; The number of folds F for cross validation; Kernel function **RBF** of SVR algorithm; **Step 1**: Establish quantitative analysis model of source domain based on Resnet1D, and the weights **W** of the layers in the model are saved. **Step 2**: Load the source domain model weights **W**, and train the model of target domain based on Resnet1D. For the source and domain models, freeze the top **L** convolutional layers. After training model, the bottleneck features ${\text{X}}_{\text{bs}}^{\text{i}}(\text{i}=1,2,\ldots ,\text{m})$ and ${\text{X}}_{\text{bt}}^{\text{i}}(\text{i}=1,2,\ldots ,\text{n})$ (Output from the flatten layers in each model) are provided as output to the SVR regressor. **Step 3**: The penalty factor C and kernel parameter gamma of the hyperparameters are optimized by the PSO algorithm and input to the SVR regressor. **Step 4**: Initialize the weight vector ${\text{w}}_{1}^{\text{i}}$ of distribution for ${\text{X}}_{\text{bs}}^{\text{i}}$ and ${\text{X}}_{\text{bt}}^{\text{i}}$ :
$$w_{1}^{i}=\frac{1}{m+n},for1\leq i\leq m+n$$
Aggregate the bottleneck features ${\text{X}}_{\text{bs}}^{\text{i}}$ and ${\text{X}}_{\text{bt}}^{\text{i}}$ into T. For t=1, …, K (step number): a. Call AdaBoost.R2 with **T**, ${\text{w}}_{t}^{\text{i}}$ , **N**, **C** and **gamma** to obtain the Model **SVR_t_**. Analogically, F-fold cross validation is used to calculate the loss **error_t_** of **SVR_t_** b. Call SVR with **T** and weight vector ${\text{w}}_{t}^{\text{i}}$ . c. Calculate the adjusted error ${\text{e}}_{t}^{\text{i}}$ for each instance as AdaBoost.R2. d. Update the weight distribution:
$$w_{t+1}^{i}=\left\{ \begin{array}{l} w_{t}^{i}\beta_{t}^{e_{t}^{i}}/Z_{t},\;1\leq i\leq n\\ w_{t}^{i}/Z_{t},\;1\leq i\leq m+n \end{array}\right.$$
where *Z_t_* is a normalizing constant, and *β_t_* is designated such that the total weight of ${\text{X}}_{\text{bt}}^{\text{i}}$ (final m) instances is:
$$\frac{m}{\left(m+n\right)}+\frac{t}{\left(K-1\right)}\left(1-\frac{m}{\left(m+n\right)}\right)$$
**Return SVR_t_**, where *t* = arg min*_i_* error*_i_*. Output: The ensemble quantitative analysis model **SVR_t_**.

The overall Resnet1D-SVR-TrAdaBoost.R2 assembles the above three models and combines their advantages to enable more accurate predictions on source and target domain datasets. The schematic diagram of Resnet-SVR-TrAdaBoost.R2 is shown in **Figure 2**. Decision tree (DF), which is often used as a learning algorithm in TrAdaBoost.R2, is replaced by a more suitable SVR. The input to SVR is provided by the bottleneck features (Output from the flatten layer) extracted by the pretrained model of Resnet. The algorithm details of Resnet-SVR-TrAdaBoost.R2 are shown in **Table 1**.

It is worth mentioning t hat there are two parameters that have a great influence on the generalization ability of Resnet1D-SVR-TrAdaBoost.R2 and need to be tuned. One is the number of calibration samples (M) in the target domain. A large number of calibration samples in target domain can improve the performance of the model, but they will also increase the learning time and cost. Hence, there is a trade-off between them. The second is the maximum number of iterations (N) of the TrAdaBoost.R2 part. Increasing N within a reasonable range can improve the robustness of the model, but overfitting will be result when it is too large. It is necessary to find a relatively suitable N, so we discussed the issue of M and N effects in detail in the following sections.

Keras (2.6.0) with Tensorflow (2.6.0) was used as the backend to implement our algorithms, running on Intel Core i7-11800H CPU at 2.30 GHz with 16 GB RAM and NVIDIA 6 GB GeForce RTX 3060 Laptop GPU.

### 2.2 Larch wood dataset

The larch samples were collected from Xinghuo Forest Farm (45°43′5.73″N, 129°13′34.37″E), Fangzheng County, Heilongjiang Province, China, which is the natural secondary forest farm of larch. Four plots on the sunny side and the shaded side were set up with a plot size of 20 m × 20 m. Three typical sample trees were selected from each plot. After each sample tree was felled, the portable chain saw was used to cut multiple wood discs continuously from the bottom to the top near the standard diameter at breast height (1.3 m at breast height). The tress were brought back to the laboratory and peeled by hand; the wood strips of 2 cm × 2 cm × 4 cm were extracted from the wooden discs with a total of 181 larch wood samples. Each sample was labeled and recorded. The samples were placed in a ventilated and dry room temperature (20°C) environment for 4 weeks, and their equilibrium moisture content was about 10%, and then the air-dry density of wood samples was determined according to the International Organization for Standardization (ISO) 13061-2: 2014 (Dahali et al., 2021).

To avoid the effects caused by surface roughness, 80-mesh sandpaper was used to polish each side of the samples five times to make the surface roughness parameter Ra close to 12.5 μm. The temperature was controlled at 20°C; the moisture content was set to 70%, 50%, 30%, and 10% in four groups; the air-dried wood samples were soaked in water for 20 days, then dried in an oven, and weighed; the moisture content of the samples was calculated every 5–15 min after drying until the moisture content of the samples was within the range of the specified variation group. When the specified moisture content value is reached, the Vis-NIR spectrum data of the samples were measured immediately. A portable spectrometer has a wavelength range of 350–2500 nm and composed of 2,151 data points; ASD LabSpec^®^ Pro FR/A114260 was used to measure the spectrum. A fiber optic probe was used to scan one time each at two different positions on the cross-section of the sample, and each scan time was about 1.5 s. The samples were continuously scanned 30 times during the set scan period. The average of the two measurements was taken as the original spectral data.

**Figure 3:**
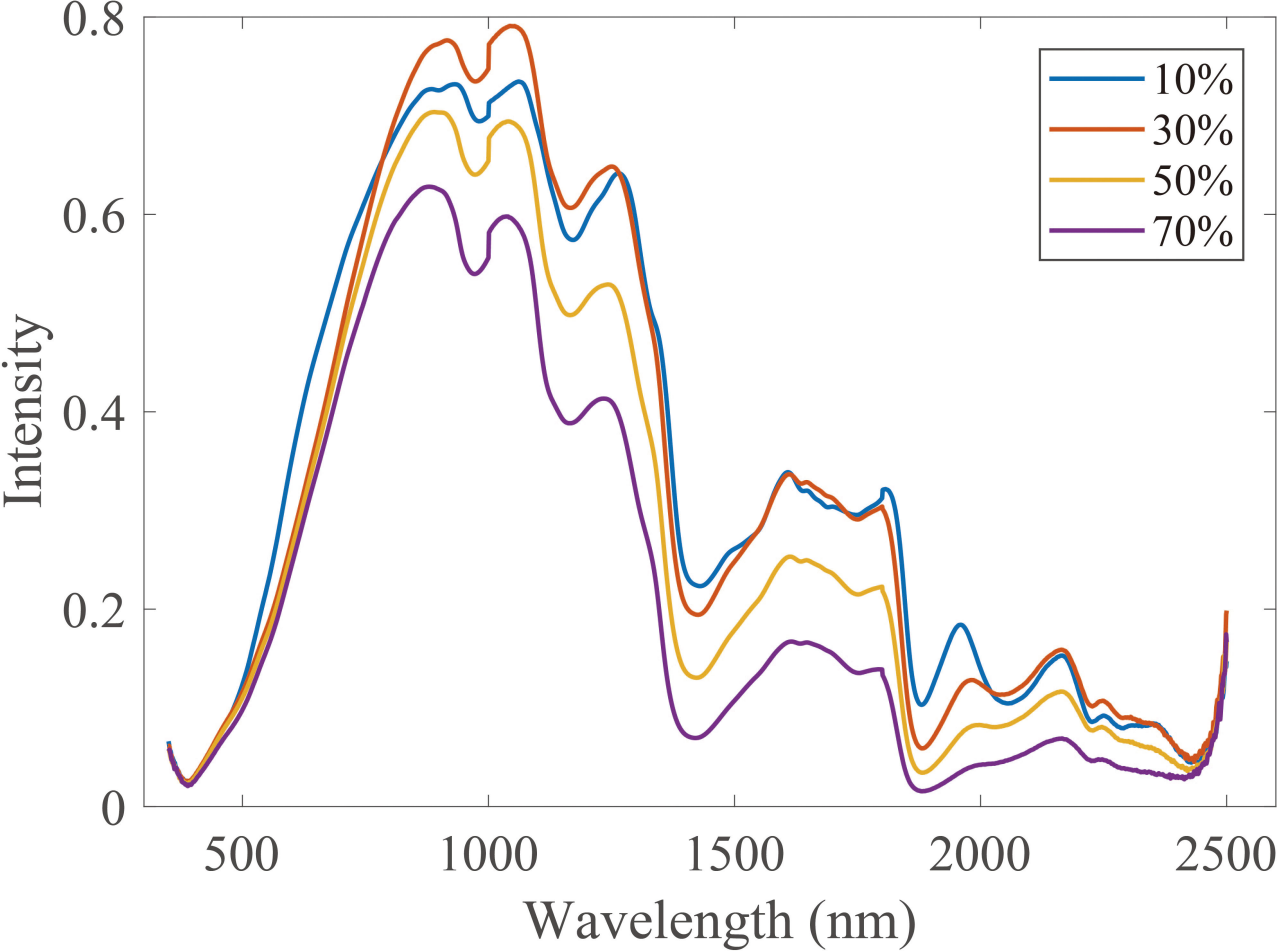
Vis-NIR spectra for wood samples with different moisture content (10%, 30%, 50%, and 70%). Vis-NIR, visible–near-infrared.

The internal structure of wood samples is varied with moisture content, which results in different spectral distributions, such as baseline shift, a small part of the absorption peak shift, and absorption peak shape change, but the overall trend of the spectra is similar (**Figure 3**). In this study, the spectral data for wood samples with 10% moisture content were used as the source domain dataset, and the spectral data for wood samples at other moisture content levels (70%, 50%, and 30%) were used as the target domain datasets. The calibration transfer was investigated in terms of the measuring environment.

### 2.3 Preprocessing of spectral data

**Figure 4:**
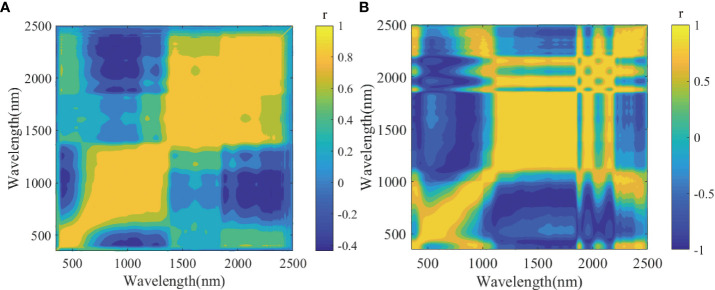
Two-dimensional correlation spectra of wavelengths for different spectral preprocessing. **(A)** Null. **(B)** SGS+SNV. r is the correlation coefficient to evaluate correlations between wavelength variables. SGS, Savitzky–Golay smoothing; SNV, standard normal variate.

Through an extensive literature review, a combination of two spectral transformation methods was selected for the preprocessing of original wood spectra (null). The 21-point Savitzky–Golay smoothing (SGS) algorithm was used to eliminate noises (Xu et al., 2021), and then the influence of particle sizes and scattering on the spectra of the sample surfaces were eliminated by combining standard normal variate (SNV) correction (Li et al., 2020b). We also compared the synchronous two-dimensional (2D) correlation spectra (Zhang et al., 2021) of wavelengths before and after the preprocessing (**Figure 4**). It is shown that the correlation between wavelengths after preprocessing (SGS+SNV) is stronger than that before preprocessing (null) significantly, which indicates that the original spectrum has more redundant information unrelated to wood density and starker collinearity, and preprocessing can improve the quality of spectral. This result is consistent with Li’s finding (Li et al., 2020c).

**Figure 5:**
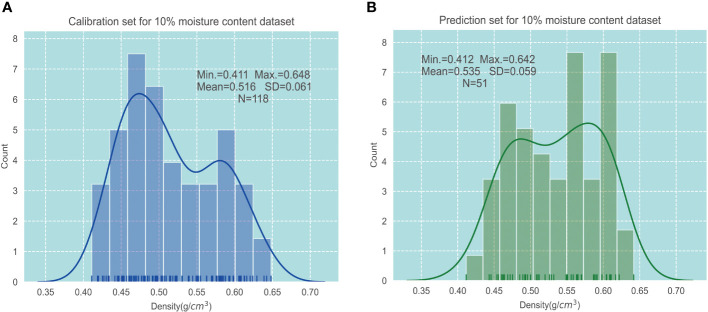
Descriptive statistics of wood density in 10% moisture content dataset. **(A)** Calibration set. **(B)** Prediction set.

In addition, the high leverage value combined with the studentized residual t-test method (Xie et al., 2017) was used to screen the singular sample numbers of the four moisture content groups in the larch wood dataset. Four groups of outlier numbers were merged into one, and the sample data corresponding to the serial number of the four groups of data were removed. Finally, 12 samples (Nos. 4, 6, 27, 39, 40, 48, 44, 45, 57, 68, 97, and 154) were eliminated, and a total of 169 samples of larch wood were obtained. The sample set partitioning based on the joint x–y distances (SPXY) method (Xu et al., 2019) was used to divide the four groups of datasets into the correction set and prediction set. Among them, the calibration set and prediction set had 118 and 51 samples, respectively. For concision, the statistical result when wood density in the 10% moisture content dataset was demonstrated (**Figure 5**), and we found that the other three “calibration-prediction” group pairs had similar results. It can be found that both the calibration set and prediction set are in normal distribution, and the mean value, standard deviation, and range of wood density in both datasets are similar, demonstrating that the division result can represent the overall distribution.

## 3 Results

### 3.1 Effects of the number of calibration samples in the target domains for calibration transfer

The purpose of the calibration transfer method is to improve the performance of the target domain model with as few calibration samples of the target domain as possible. Therefore, it makes sense to determine an appropriate range of calibration sample sizes in the target domain. In this study, the number of calibration samples for different target domains (M) was set as 20, 40, 60, 80, 100, 120, and 140. The SPXY method was used to collect the calibration and prediction samples of the target domains to ensure the representativeness of distribution for each moisture content group. Among them, the predicted sample size was set to 30 for both source and target domains, and the calibration samples are selected from the remaining samples.

For the proposed Resnet1D-SVR-TrAdaBoost.R2, a robust source domain model (10% moisture content group) was first constructed. The 118 calibration samples selected in Section 2.3 were used to train the Resnet1D model, and *R* ^2^ and root mean square error (RMSE) of the prediction were used to evaluate the generalization ability. To remove the effect of random parameters in the CNN, the finalized model (*R* ^2^ = 0.7174, RMSE = 0.0312) was the one that was closest to the mean (*R* ^2^ = 0.7145, RMSE = 0.0318) of 20 repetitions of training. Next, the weights Ws of the source domain model were saved and loaded into the target domain model as a pretrained model. The first 10 convolutional layers were frozen to fine-tune the weights of the target domain model, and then, the bottleneck features after the flattening layer were imported into the SVR regressor. The maximum number of iterations (N) was set to 50.

**Figure 6:**
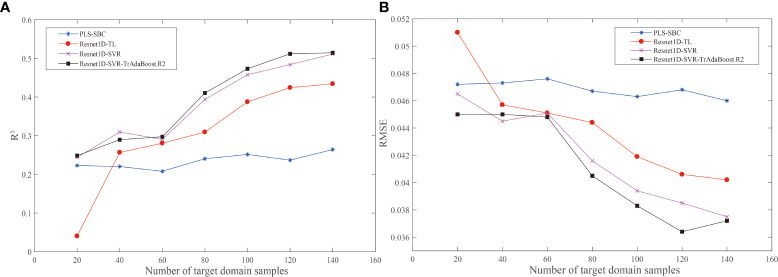
Effects of the number of target domains for calibration transfer. **(A)** *R* ^2^. **(B)** RMSE. RMSE, root mean square error.

To verify whether Resnet1D-SVR-TrAdaBoost.R2 method is effective and whether it is better than traditional methods, we added PLSR+SBC (partial least squares regression (PLSR) model transferred by SBC method), Resnet1D-TL (Resnet1D model based on transfer learning), and Resnet1D-SVR (Resnet1D-SVR model based on transfer learning) in this protocol for comparison. The proposed Resnet1D-SVR-TrAdaBoost.R2 was used as the calibration transfer method; the experiments in the target domain groups with a wood moisture content of 70%, 50%, and 30% were implemented; and the results of three groups were averaged (**Figure 6**). It is worth mentioning that the results in each group were the average of 15 times running, to overcome the impacts of random parameters. There is no doubt that the obtained results are the least desirable when the target domain data are used to train the model directly, so no comparison is made here.

In **Figure 6**, as the sample size of the target domain increased, the performance of the models improved gradually. All models achieved the best predictions at 140 samples. When M was 20, the performance of Resnet1D-TL (*R* ^2^ = 0.0404, RMSE = 0.051) was the worst; it implied that the calibration ability of deep transfer learning was poor when there were few samples in the target domain. When M was greater than 40, the prediction effect of the PLSR+SBC model was the worst, which means that even if the target domain samples were sufficient relatively, the calibration ability of the deep transfer learning-based methods was still stronger than the traditional calibration transfer methods, and with the increased of samples, the gap was widening. The trend of Resnet1D-SVR-TrAdaBoost.R2 and Resnet1D-SVR was similar, but the performance of Resnet1D-SVR-TrAdaBoost.R2 was better, which shows that TrAdaBoost.R2 was necessary to calibrate the parameters. It is worth mentioning that there is an exception here; when M was 40, the prediction effect of Resnet1D-SVR (*R* ^2^ = 0.3095, RMSE = 0.0445) was better than that of Resnet1D-SVR-TrAdaBoost.R2 (*R* ^2^ = 0.2897, RMSE = 0.0450). Comparing the result data, we found that the model evaluation metrics (*R* ^2^ and RMSE) of Resnet1D-SVR fluctuated greatly during the repeated experiments, and the prediction effect was not stable enough, so the high average result was accidental. When M was greater than 60, Resnet1D-SVR-TrAdaBoost.R2 had the absolute advantage of accuracy in target domain samples.

### 3.2 Performance comparison of models built by different calibration transfer methods

**Figure 7:**
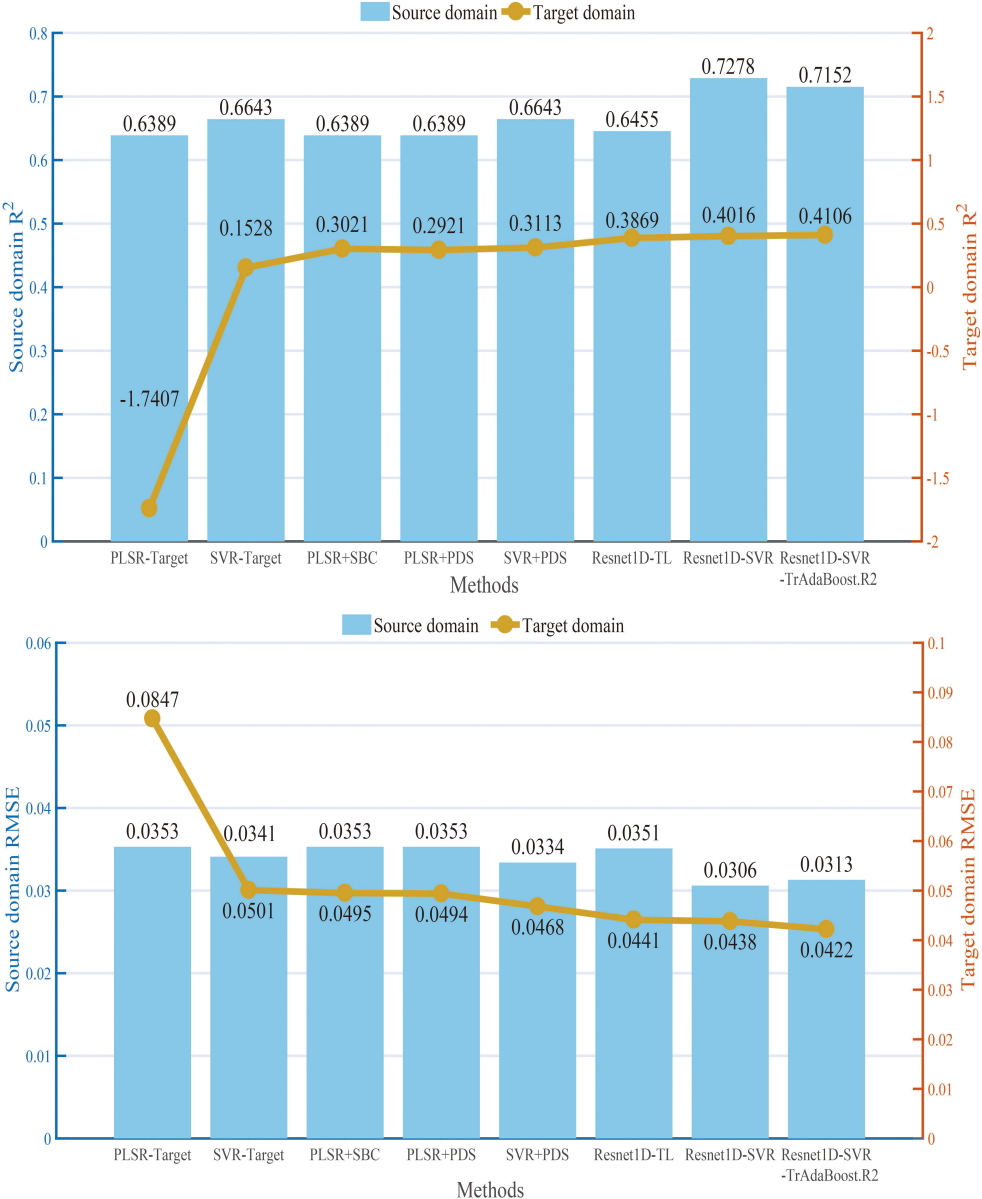
*R* ^2^ and RMSE values of different calibration transfer methods in source and target domains. RMSE, root mean square error.

In this subsection, the performance of models built with different calibration transfer methods was compared, and the calibration capabilities of Resnet-SVR-TrAdaBoost.R2 were discussed. In this protocol, the calibration and prediction samples selected in Section 2.3 were used to test the methods, and the weight Ws of the source domain model was the same as described in Section 3.1. PLSR and SVR without any calibration transfer (PLSR-Target, SVR-Target) were chosen as a comparison. PLSR+SBC, PLSR+PDS (PLSR model transferred by PDS method), SVR+PDS (SVR model transferred by PDS method), Resnet1D-TL, and Resnet1D-SVR were chosen as baselines. For the proposed Resnet1D-SVR-TrAdaBoost.R2, the maximum number of iterations (N) was set to 50. The experiments with a wood moisture content of three groups were implemented in the target domain sample sets, and the average results were presented (**Figure 7**). It is worth mentioning that the results in each group were the average of 20 times running and overcame the impacts of random parameters.

As shown in **Figure 7**, the non-linear method (SVR) had a much better performance in both source and target domains as compared with the linear method (PLSR). In the traditional calibration transfer method based on PLSR, the calibration ability of SBC has an outstanding performance (*R* ^2^ = 0.3021, RMSE = 0.0495). The prediction ability of SVR+PDS (*R* ^2^ = 0.3113, RMSE = 0.0468) was the best among non-transfer learning methods, especially since the *R* ^2^ value of SVR+PDS in the source domain was 0.0188 higher than Resnet1D-TL, but Resnet1D-TL performed better in the target domain. Overall, the prediction accuracy of the models built by the transfer learning method was higher. Among them, a strong generalization ability of Resnet1D-SVR-TrAdaBoost.R2 was exhibited in both the source domain (*R* ^2^ = 0.7152, RMSE = 0.0313) and the target domain (*R* ^2^ = 0.4106, RMSE = 0.0422). The performance of the prediction model was the best among all methods in the target domain.

### 3.3 Performance of calibration transfer models for different larch wood moisture content

Air-dry density is a strength indicator, which is often used in the production and circulation of wood. Moisture content and density of wood are related closely. If the actual moisture content is lower than the equilibrium moisture content, moisture hygroscopicity of wood will be exhibited; otherwise, moisture evaporation of wood will be exhibited. Therefore, it is essential to establish a model that can predict the air-dry density of wood in different moisture contents. In this subsection, the calibration transfer between different moisture contents was investigated. PLSR was used to establish the prediction models of each moisture content group and used as a standard. PLSR-Target was used as a reference, and the proposed Resnet1D-SVR-TrAdaBoost.R2 was used to calibrate. Calibration and prediction samples were the same as in Section 2.3. The number of N is 50.

**Figure 8:**
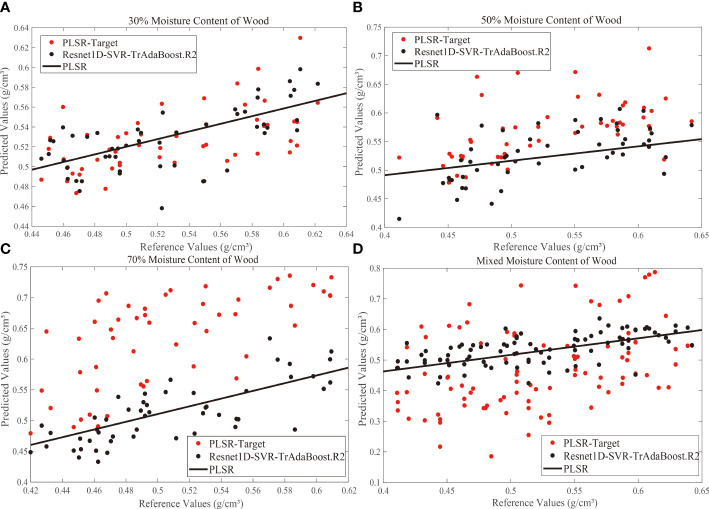
Correlation between standard test values and Vis-NIR predicted values derived from calibration transfer models for 30% **(A)**, 50% **(B)**, 70% **(C)**, and mixed **(D)** moisture content. Vis-NIR, visible–near-infrared.

In the actual measurement, there are individual differences in the moisture content of a batch of wood. Therefore, we added a new experimental group, and the SPXY method was used to select 40 samples from each target domain experimental group (moisture content of 30%, 50%, and 70%), and these samples were merged into a calibration set with 120 samples. Similarly, 30 samples were selected from the remaining samples and merged into a prediction set with 90 samples. The calibration transfer results are shown in **Figure 8**.

The above results indicated that the scatter points of the predicted values (PLSR-Target) with 50% and 70% moisture content were above the PLSR predicted line mostly, and the overall trend of the predicted value was large. Most of the scatter points of the predicted values with 30% moisture content were located near the PLSR prediction line, which means that as the moisture content increased, the hygroscopic effects of the woods were enhanced, and the free water in intracellular was also increased. The increase of moisture content and the change of internal structure could interfere with the Vis-NIR spectrum seriously during the hygroscopicity of wood, which generated the poor prediction effect of the model. Different moisture contents affect the response function, and large systematic errors will occur when the 10% moisture content model was used to predict spectra under other moisture content conditions.

After calibration transfer by Resnet1D-SVR-TrAdaBoost.R2, it can be seen that the predicted scatter points of the 30%, 50%, and 70% moisture content groups were close to the PLSR line intuitively, while the scatter points of the mixed moisture content group were relatively close. This experiment showed that Resnet1D-SVR-TrAdaBoost.R2 had a robust generalization ability even though the spectra were affected by the detection environments greatly, and it had the potential for practical application for different water content or mixed water content.

## 4 Discussions

The above experiments have proved that the prediction approach of larch wood density from Vis-NIR spectroscopy based on parameter calibrating and transfer learning (Resnet1D-SVR-TrAdaBoost.R2) proposed in the present study had a great generalization ability in calibration transfer. The advantages and limitations of this hybrid method would be discussed from three aspects including model performance, the effect of the maximum number of iterations (N) on modeling, and the practical application of the model in forestry production.

### 4.1 Comparison of model predictive ability

**Figure 9:**
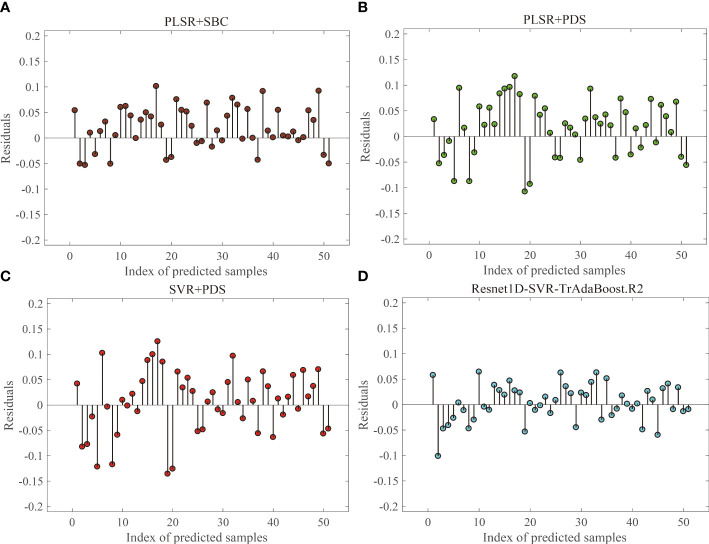
Residual analysis for target domain with 70% moisture content group: PLSR+SBC **(A)**, PLSR+PDS **(B)**, SVR+PDS **(C)**, Resnet1D-SVR-TrAdaBoost.R2 **(D)**.

For the prediction results in Section 3.3, residual plots (**Figure 9**) were used to compare and analyze the applicability and residuals of the proposed Resnet1D-SVR-TrAdaBoost.R2 with other calibration transfer methods. For concision, the results when the target domain was 70% moisture content group were shown, and the results for other groups were similar. The four residual values fell on both ends of the 0-axis evenly, proving that the prediction values of the four methods are distributed equally. The prediction values within the range of ±0.15 have strong interpretability, which proves that the prediction model has strong reliability. The residuals of Resnet1D-SVR-TrAdaBoost.R2 were smaller than those of PLSR-PDS and SVR-PDS significantly, and prediction values of Resnet1D-SVR-TrAdaBoost.R2 had extreme interpretability in the range of ±0.1. The performance of PLSR-SBC was between Resnet1D-SVR-TrAdaBoost.R2 and the other two methods; the results were consistent with the results of **Figure 7**.

Currently, traditional calibration transfer methods (e.g., SBC and PDS) attempt to minimize data differences in sample sets or target values and thus use the master model to make predictions about the properties of slave data, and most new algorithms are proposed based on this underlying principle (Fan et al., 2008; Du et al., 2011; Workman, 2018), while others seek an explicit feature space transformation that maps the spectra of the source and target domains into a space orthogonal to the interfering factors (Zhu et al., 2008; Igne et al., 2009; Das et al., 2012). All of these methods require the support of a large amount of data to discover similar patterns between different data domains. At the same time, the quality of the data can cause large interference with the above methods, which is why numerous spectral preprocessing methods (Zhen et al., 2008) and feature band selection methods (Fu et al., 2022) are proposed to reduce the interference as much as possible, which requires a large number of comparison experiments, and the cost of model application is increased. The proposed hybrid model has a feature extractor, which can exclude the interfering bands in the training and reduce the dependence of the model on the quality of the original data; meanwhile, the depth model can learn the underlying information in the data during the training process, which reduces the demand of the model on the sample size; the introduction of the fine-tuning and TrAdaBoost.R2 methods makes it have a certain self-renewal capability. Comprehensive analysis shows that this hybrid model is better than the traditional calibration transfer methods.

### 4.2 Effects of maximum iterations on model performance

**Figure 10:**
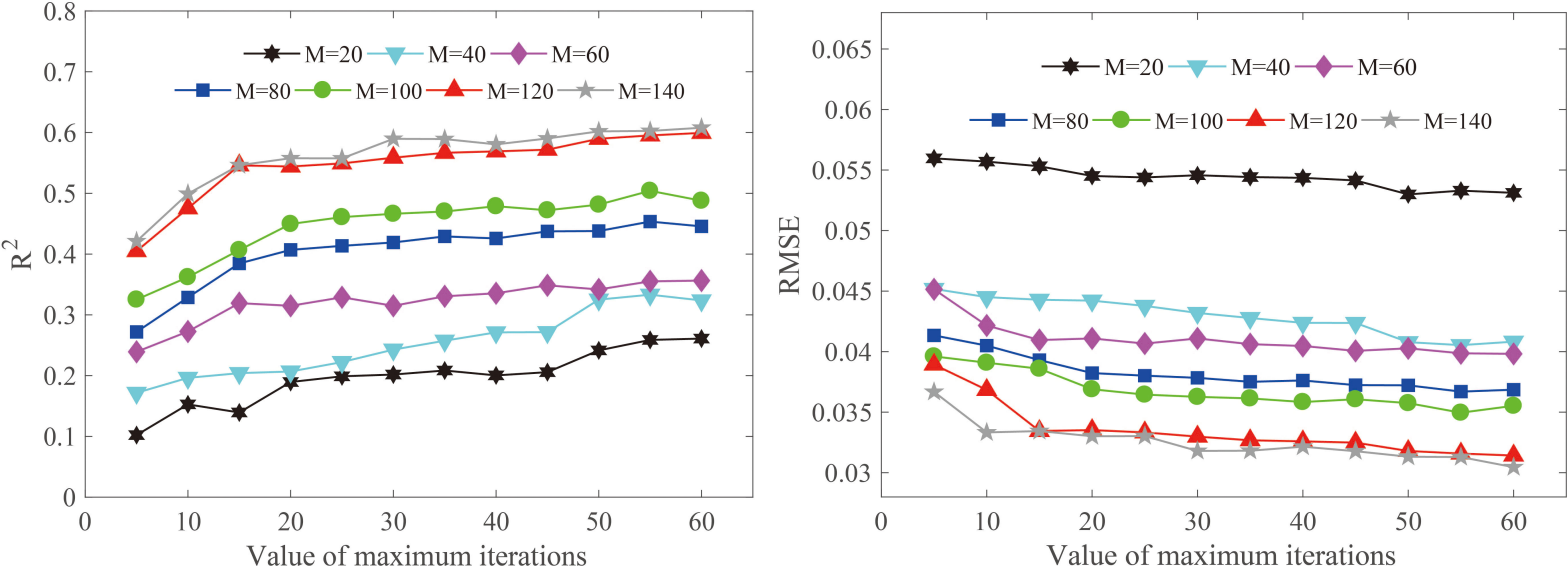
Effects of the value of maximum iterations on model performance.

The proposed model of Resnet1D-SVR-TrAdaBoost.R2 was established based on AdaBoost.R2 strategy. The performance of the model was affected by the maximum number of iterations (N). If N was too small, the calibration effect of the model was unsatisfactory; otherwise, the complexity and computing time of the algorithm were increased. Therefore, we explored the impact of N on the generalization performance of the model. For concision, the effects when the target domain was 30% moisture content group were shown (**Figure 10**), and the results for other groups were similar. The trends of the evaluation metrics *R* ^2^ and RMSE were similar, and when the number of samples (M) in the target domain calibration set increased from 20 to 140, the trends were almost the same. This means that increasing the value of N could improve the generalization performance of the Resnet1D-SVR-TrAdaBoost.R2 model significantly. When the number of N was greater than 50, the performance of the model tended to be stable, so the number of N was set as 50 in this study. In practical applications, it is recommended to set the number of N to be greater than 30.

### 4.3 Practical application in forestry industries

The results show that Resnet1D-SVR-TrAdaBoost.R2 has good generality and accuracy, but some limitations also need to be noted.

In practical applications, the measurement cost of target domain calibration samples (M) in calibration transfer is high. For the air-dry density prediction of wood under different moisture contents, volume measurement, drying, and weighing of wood were required, which were costly and time consuming. In forestry industries, a huge part of the manpower and material resources are consumed in the measurement of many wood properties. Therefore, it is necessary to reduce the need for labeling samples. The proposed hybrid method could reduce the demand for measured samples of the target domain to a certain extent. By comparing the performance of models built with different numbers of M, it could be seen that Resnet1D-SVR-TrAdaBoost.R2 still fails to achieve good prediction accuracy when M was less than 80, but the performance of the model could reach a satisfactory level when M was larger than 80 (as shown in **Figure 6**).

Overall, the performance of Resnet1D-SVR-TrAdaBoost.R2 hybrid method was better than that of other methods. Therefore, in actual production, if the requirements for prediction accuracy are high, Resnet1D-SVR-TrAdaBoost.R2 will be the optimal choice. In addition, the specific number of recommendations for M may be instructive for the application of transfer learning techniques in practical forestry. At the same time, scientific and standardized field sampling is recommended to ensure the representativeness of labeling samples.

The proposed hybrid algorithm requires iterative training, and Vis-NIR spectral data have many characteristic variables, so the training time of the algorithm is long, and the computing capacity of the device is required to be higher. During the experiments, the running time of Resnet-SVR-TrAdaBoost.R2 was about 30 s, while the running time of SBC and PDS was only 1–2 s. In actual production, if fast detection speed is required without much high accuracy, the traditional calibration transfer methods can be satisfied. However, if higher prediction accuracy is required, then Resnet-SVR-TrAdaBoost.R2 will be a satisfactory choice.

It is worth mentioning that the prediction ability of Resnet1D-SVR-TrAdaBoost.R2 was the best when the difference in target and source domain distributions was larger (as shown in **Figures 7**, **8**). Compared with the traditional deep transfer learning algorithm, the prediction performance of Resnet1D-SVR-TrAdaBoost.R2 was more stable and accurate, but more parameters in the training process were needed to train and required more sample size and training time. Furthermore, although Resnet-SVR-TrAdaBoost.R2 was validated under different measurement conditions, validation under other tree species was incomplete. Therefore, the proposed hybrid method needs to be further tested for its applicability to other species.

## 5 Conclusion

The problem of low optimization performance of traditional calibration transfer methods when there are significant non-linear differences between the spectra of different measurement environments was addressed. A deep transfer learning strategy (Resnet1D-SVR-TrAdaBoost.R2) based on TrAdaBoost.R2 parameter calibrating and SVR feature optimization was proposed in this study. The method was fully analyzed, verified against field observations, and compared with conventional calibration transfer methods.

The experimental results showed that the proposed hybrid method had a good performance. When predicted with larch wood air-dry density in different moisture contents, the spectra of the high-dimensional and non-linear were extracted by the proposed method. The non-linear differences between source and target domains were weakened by SVR, and finally, the parameters of each sample were calibrated by TrAdaBoost.R2. In terms of prediction accuracy, the prediction accuracy of the proposed hybrid method was superior to other methods (source domain: *R* ^2^ = 0.7152, RMSE = 0.0313; target domain: *R* ^2^ = 0.4106, RMSE = 0.0422). In terms of demand for calibration samples of the target domain, the performance of the proposed hybrid method (M > 80) was superior to the traditional transfer learning strategy (M = full calibration samples), also better than Resnet1D-SVR (M > 90).

Furthermore, the satisfactory prediction accuracy could be obtained by a proposed hybrid method when the source domain was different from the target domain. In addition, the hybrid strategy used for the density retrieval of larch wood also performed well in the density inversion of larch wood in mixed moisture content. A limitation is that compared to traditional calibration transfer strategies, the method had a longer running time, and its requirements for the calculation capacity of the equipment were higher. By comprehensive consideration, all the results indicated that Resnet1D-SVR-TrAdaBoost.R2 performed well with high versatility, accuracy, and portability in density inversion of larch wood and was an accurate and feasible method.

## Data availability statement

The original contributions presented in the study are included in the article/supplementary material. Further inquiries can be directed to the corresponding author.

## Author contributions

ZZ contributed to the draft writing and editing. YXL contributed to the method design, supervision, and experimental design. YL contributed to the visualization. All authors contributed to the article and approved the submitted version.

## Funding

This research was funded by the Fundamental Research Funds for the Central Universities (grant number 2572022AW45) and the Applied Technology Research and Development Plan of Heilongjiang Province (grant numbers GA19C006, GA21C030).

## Acknowledgments

The authors would like to thank the editor and reviewers for their constructive comments.

## Conflict of interest

The authors declare that the research was conducted in the absence of any commercial or financial relationships that could be construed as a potential conflict of interest.

## Publisher’s note

All claims expressed in this article are solely those of the authors and do not necessarily represent those of their affiliated organizations, or those of the publisher, the editors and the reviewers. Any product that may be evaluated in this article, or claim that may be made by its manufacturer, is not guaranteed or endorsed by the publisher.
